# Clinical trial simulation of antiviral drugs

**DOI:** 10.1128/jvi.01814-24

**Published:** 2026-04-17

**Authors:** Joshua T. Schiffer, Daniel B. Reeves, Bryan Mayer, Lucero Rodriguez Rodriguez, Elizabeth R. Duke, Beatrix Haddock, Ugo Avila-Ponce de Leon, Shingo Iwami, Katherine Owens, Shadi Esmaeili-Wellman

**Affiliations:** 1 Fred Hutchinson Cancer Center, Vaccine and Infectious Diseases Division 7286, Seattle, Washington, USA; 2 Division of Allergy and Infectious Diseases, University of Washington 7284, Seattle, Washington, USA; 3 Department of Global Health, University of Washington 7284, Seattle, Washington, USA; 4 Palo Alto VA, Palto Alto, California, USA; 5 University of California, San Francisco 8785, San Francisco, California, USA; 6 Interdisciplinary Biology Laboratory (iBLab), Division of Biological Science, Graduate School of Science, Nagoya University 12965, Nagoya, Japan; Indiana University Bloomington, Bloomington, Indiana, USA

**Keywords:** clinical trial, antiviral drugs, mathematical modeling

## Abstract

Antiviral clinical trial simulation (CTS) is a type of mathematical modeling that couples viral- immune dynamics (VID) unique to each human viral pathogen, with mechanistic, pharmacokinetic (PK), and pharmacodynamic (PD) drug characteristics. Validation is achieved by matching model output to detailed viral load trajectories from trials. Antiviral CTS can be applied at all stages of drug development to viruses with distinct shedding patterns. Models can capture the activity of small molecules, neutralizing antibodies, and cellular therapies, as well as combination strategies to enhance potency and avoid drug resistance. Several principles are observed across antiviral CTS models. First, PK and PD models that recapitulate drug levels and concentration-dependent antiviral activity are often necessary, but never sufficient to predict trial results. VID equations are also required to guide optimal treatment timing because expanding immune responses synergistically eliminate infection but are deleterious if too sustained or intense. Therefore, equivalent antiviral doses may have different efficacy if given during different infection stages. Second, antiviral CTS models identify effective plasma drug concentrations in humans, which are often poorly predicted by *in vitro* assays. Finally, models that do not consider drug mechanisms lead to incorrect efficacy estimates. Data-validated CTS is increasingly used to inform drug dose and dosing interval, treatment timing and duration, virologic endpoint selection, and sample size, particularly when applied to detailed phase 1 and 2 trial data. Given the high expense of antiviral licensure trials, CTS models are vital to optimize trial efficacy and de-risk the drug development process.

## INTRODUCTION

Quantitative systems pharmacology (QSP) is a computational modeling approach that integrates disease processes and drug characteristics to inform the design and conduct of clinical trials (1). The goal of QSP is to predict trial outcomes under various untested treatment assumptions. The demand for predictive trial analytics is rapidly expanding. In 2025, the QSP market was estimated at USD 1.07  billion and forecasted to grow to USD 3.22 billion by 2033. More than 350 QSP-containing submissions were made to the Federal Drug Administration (FDA) across medical sub-specialties, with 80 in 2023 (2). With accelerating regulatory adoption and impact, QSP is recognized as a scalable and efficient method to inform key details of trial design, particularly drug dosing strategies (2, 3).

Clinical trial simulation (CTS) of antiviral drugs is unique within the QSP research space because antiviral CTS models are tested, not only against metrics of drug levels and drug potency but also against individualized, highly granular, longitudinal, and quantitative viral loads, which serve as dynamic biomarkers of infection biomass (4–7). Antiviral CTS models can therefore often achieve a higher degree of validation than QSP models for cardiovascular, oncologic, autoimmune diseases, or other infectious diseases that are characterized by binary clinical outcomes, or by less specific and less frequently sampled biomarkers. There is an opportunity for antiviral CTS to inform modeling strategies of other diseases. For instance, quantitative serum tumor biomarkers are increasingly used in the clinic and have been usefully modeled for chronic myelogenous leukemia (CML) (8, 9), prostate cancer (10), and ovarian cancer (11). However, it is still rare for these measures to be obtained daily in cancer clinical trials, as is typical for viral infections.

The non-linear nature of longitudinal viral load, immune response, and treatment data during infection necessitates mathematical models that encode precise mechanisms, which couple viral replication and spread in tissues with innate and acquired immune responses during therapeutic interventions (12). Thus, in addition to informing practical matters of trial design such as drug, dose, dosing interval, treatment timing, and virologic endpoint selection, antiviral CTS improves mechanistic understanding of how treatments directly and indirectly impact the course of viral infections in different populations (13). Validated CTS models often have counterintuitive results not obtainable without bridging virology, immunology, and pharmacology across molecular, cellular, and organismal scales.

**Fig 1:**
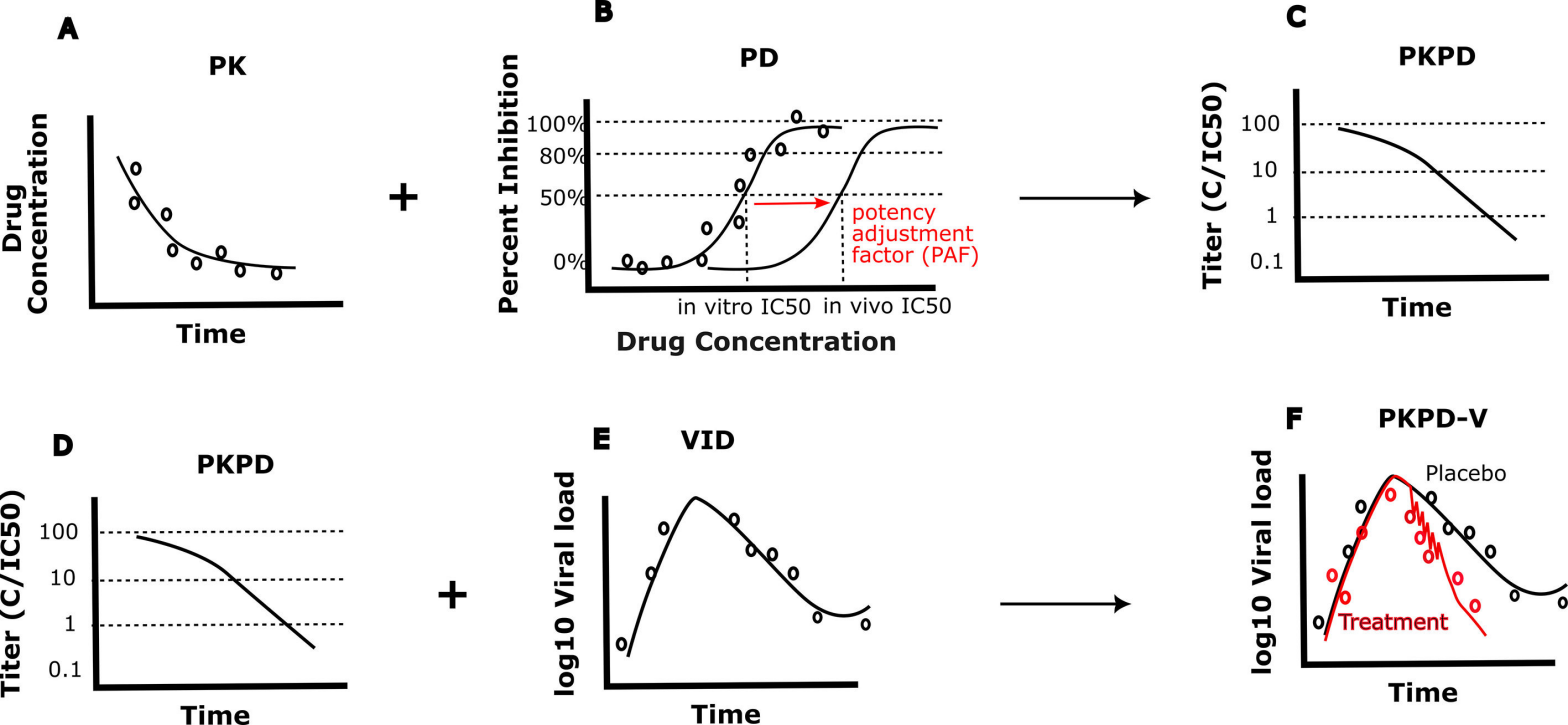
Schematic of clinical trial simulation model components. In all panels, dots represent hypothetical individualized data, while lines are model output. (**A**) PK models capture drug levels over time. (**B**) PD models correlate drug concentrations with inhibition of viral replication or infection of new cells *in vitro* but often must be adjusted when modeling clinical trial data, as required drug concentrations to achieve inhibition often differ. (**C and D**) Joint PKPD models capture the inhibition of viral replication as a function of time according to drug levels. (**E**) VID models capture the dynamics of natural, untreated infection, which are influenced by timing and intensity of immune responses. (**F**) Integrated CTS models (VID + PK + PD) quantify the effect of a drug on viral load. VID models are critical for capturing the timing and intensity of immune responses, which, along with treatment, determine the virologic outcome of infection.

Antiviral CTS increasingly consists of stepwise development of three types of models, which are validated individually and then integrated into a single equation set, which can be tested against viral load data from clinical trials (Fig. 1). Pharmacokinetic (PK) models capture drug and key metabolite levels over time. PK models traditionally focused on small-molecule agents but have expanded in scope and sophistication to include various immunotherapies and drug delivery systems (14–21). Pharmacodynamic (PD) models quantify static drug concentration-related antiviral activity and toxicity and are routinely merged with PK models to estimate antiviral potency as a function of time. Modern PD models distinguish dose-dependent additive, multiplicative, and synergistic potency effects of drug combinations and can make probabilistic assessments of emergent drug resistance (22, 23).

The foundational principle of antiviral CTS models is that PK and PD models are often necessary, but never sufficient to predict trial outcomes. Viral-immune dynamic (VID) models, which capture rates of infected cell turnover and immune responses against infected cells and viruses, are also crucial (Fig. 1). Antiviral agents work in concert with innate immune responses which correspond to contemporaneous intensity of infection (24, 25), as well as acquired memory responses which expand during infection and are then sustained for weeks to years (26, 27). The timing, phenotype, and effectiveness of immune responses vary enormously across human viral pathogens, and between different study populations with differing immune statuses. Occasionally, as described below for HIV, hepatitis B, and hepatitis C, meaningful insights can be derived from VID models alone, without explicit consideration of PKPD.

**Fig 2:**
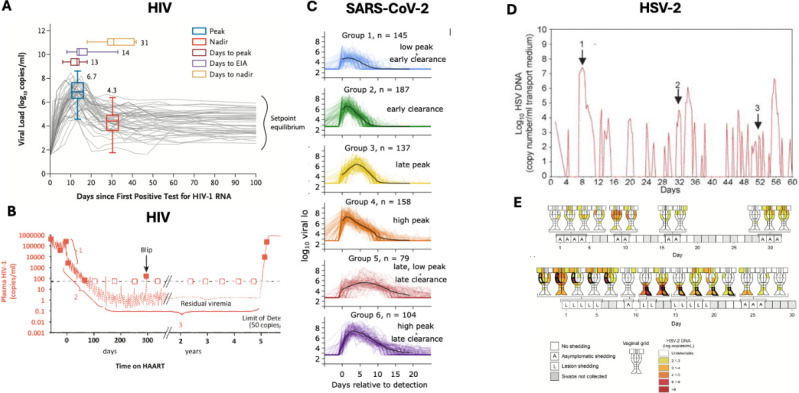
Unique viral kinetics associated with different human viral infections. Different VID model types are needed to capture viral kinetics of chronic persistent, acute self-limited, and chronic recurrent viral infections. (**A and B**) HIV is shown as an example of *chronic persistent infection*. (**A**) Data from 50 untreated primary infections show plasma viral load peaks, contracts, and then reaches a variable steady state which persists until the development of AIDS. (37) (**B**) Antiretroviral treatment induces triphasic decay and halts disease progression. (**C**) 810 SARS-CoV-2 infections are shown as examples of *acute self-limited infection* (28). Infection phenotypes based on nasal sampling can be clustered into six groups according to viral kinetic features such as peak viral load, expansion and clearance slope, and duration of viral shedding. Effective antiviral treatment (not shown) increases viral clearance slope from time of treatment as in Fig. 1F. (**D and E**) HSV-2 is shown as an example of a *chronic recurrent infection*. (**D**) Genital sampling from a single individual reveals frequent, heterogeneous episodes of shedding (38). Higher viral loads correspond with contemporaneous ulcer formation and transmission risk. (**E**) Shedding is spatially heterogeneous, stemming from dozens of concurrent micro-environments (39). Effective antiviral treatment (not shown) decreases shedding rate, episode rate, and peak episode viral load.

Each human viral pathogen has a distinct kinetic fingerprint, reflecting variability in the speed of viral replication and timing of immune responses (Fig. 2) (4, 28–34). This heterogeneity leads to different optimal treatment strategies and windows for each virus. In some cases, effective drugs that are dosed too early may have diminished efficacy due to lack of ongoing immune responses (24). Yet, when dosed too late, these same drugs may fail to prevent aberrant and harmful immune-related inflammation associated with high antecedent viral load (25, 35, 36). Viral kinetics are also highly variable among individuals with the same infection (Fig. 2). To account for these nuances and to optimize timing and duration of therapy, PK and PD models must be coupled with validated VID models.

This integrative modeling approach yields unique insights. First, antiviral CTS models are the best method to identify human plasma drug concentrations required to suppress viral infection of cells. Importantly, CTS model-derived estimates often differ markedly from *in vitro* assay projections, which guide initial dosing (27). Second, CTS models optimize timing, duration, and dosage of treatment to maximize synergy with immune responses and avoid unexpected outcomes such as viral rebound following treatment (24, 40). Third, CTS models help select virologic endpoints according to drug mechanism of action, which may alter viral clearance rate independent of potency (41, 42). These considerations are vital for power and sample size calculations for future trials. Finally, antiviral CTS models can be tested across trials with discordant results to provide meta-analysis-level results (42).

**Fig 3:**
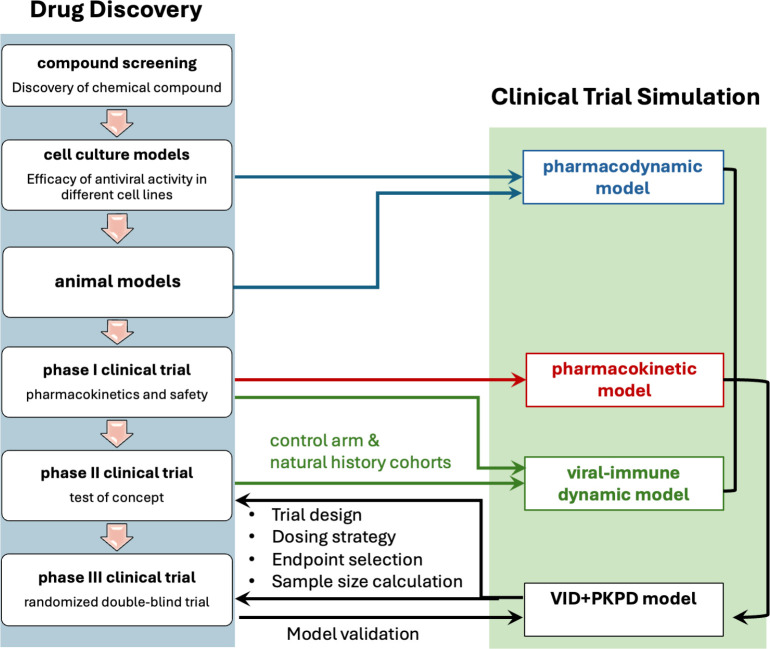
Clinical trial simulation integration with the drug development pathway. In practice, PK, PD, and VID models are often informed by different data sources and studies. Ideally, these data can be gathered from participants in phase 1 and 2 trials to allow rigorous model validation.

In this review, we discuss the high utility of antiviral CTS models throughout all phases of the drug development process, spanning pre-clinical development to phase 1–3 human trials to post-licensure analyses. We focus on data types required for each type of model development with examples at each stage. We review the array of VID, PK, and PD models that have been leveraged for antiviral CTS and highlight results showing that varying viral kinetic patterns and drug characteristics necessitate different modeling strategies. A critical theme is that integration of an experienced modeling team early during antiviral drug development can optimize all subsequent trial stages and significantly de-risk the transition from phase 1 to more expensive and demanding phase 2 and 3 trials (Fig. 3).

## VIROLOGIC SURROGATE EFFICACY ENDPOINTS AS JUSTIFICATION FOR MODELING LONGITUDINAL VIRAL LOADS

The typical trial endpoints required for licensure of an antiviral are appropriately based on clinical benefit and may include reduction in hospitalization rate, death rate, or severity or duration of symptoms (43–47). For many acute viral infections, such as SARS-CoV-2, severe endpoints only occur in a fraction of trial participants. For chronic viruses such as HIV and hepatitis B and C, clinical endpoints accrue slowly over many years (48, 49). Both of these issues necessitate trials with large sample sizes to achieve sufficient statistical power. Licensure trials are therefore costly and personnel intensive (50).

Because virologic endpoints can be assessed in *all* trial participants, serial measurement of viral load allows trials to be conducted with higher statistical power and in fewer people, which, in turn, decreases the length and cost of the trial (51–53). A virologic outcome must be formally established as a statistical surrogate of clinical benefit before it can be used as a primary trial endpoint using a set of pre-established criteria (54). Selection of virologic surrogate endpoints relates directly to a pathogen’s kinetic pattern and has included plasma viral suppression for HIV-1 (55), hepatitis B (5, 56), hepatitis C (57, 58), and cytomegalovirus (CMV) (59); viral clearance slope for SARS-CoV-2 (51, 60) and Ebola (61); and reduction in mucosal shedding rate for herpes simplex virus-2 (HSV-2) (62). These surrogate endpoints are established and accepted to varying degrees for each of these viruses. Moreover, there has yet to be an example where a virologic endpoint could *not* be identified for a human virus. It is therefore plausible that all human viruses have a virologic surrogate under treatment.

The consistent identification of viral load as an accurate biomarker of disease burden validates modeling viral load as a useful method to assess therapeutic potency. Models might also be used to identify highly sensitive virologic endpoints for future trials. For instance, in mechanistic VID models, viral area under the curve corresponds directly to the number of infected cells, which, in turn, is a representation of the surface area of virally mediated tissue damage (34, 63). Importantly, while viruses replicate and spread exponentially, tissue damage is accrued on a linear scale such that even a 0.5 log reduction in viral load maps to a greater than 75% reduction in biomass of infected tissue (63). This likely explains why relatively small reductions in viral load during treatment of SARS-CoV-2 and Ebola corresponded with substantial clinical benefit (43–45, 61), and why only partial suppression of HIV viremia, while not ideal, considerably slows progression to AIDS and death (64, 65).

## VIRAL LOAD DATA FOR VID MODELS

The goal of VID model development is to accurately recapitulate serial viral loads during untreated infection with a set of biologically realistic equations. Human viral load data come from prospective cohort studies intended to capture the natural history of disease (66), or from clinical trials’ placebo arms. VID models can also be designed for pre-clinical animal models of infection to inform early studies in humans (67).

Viral load is usually assessed with quantitative polymerase chain reaction (qPCR). While qPCR for both RNA and DNA viruses detects mostly non-viable viral genomes, it has high utility for modeling because it is extremely sensitive and reproducible (34). Given the rapid degradation of viral genomic material outside its host cell, a sample with detectable viral genomic material likely indicates recent viral genome amplification within an infected cell or persistence of latent viral forms (68). Some models are fit separately or jointly to quantitative viral culture data (69). Yet, with the exception of influenza, these data are less commonly available (70). Quantitative viral culture assays are less sensitive and precise than qPCR, which limits the number of datapoints for fitting and lowers the veracity of data fitting. In some cases, assays that detect intact genomes or encapsulated viral forms can balance sensitivity and specificity for replication-competent virus (71, 72).

Assay characteristics can profoundly impact observed trial and modeling outcomes. The viral dynamic range in a trial is defined as the difference between the viral load at the time of enrollment and the assay’s lower limit of detection (LOD) (42). A narrow dynamic range, which may be caused by late enrollment, lower genomic amplification with PCR, or a high assay LOD, can limit observed antiviral effect despite a meaningful reduction in infection burden. Dynamic range variability must be considered as one of several possible explanations when trials with similar populations testing the same drug yield conflicting results (42). Reliable assays that limit noise also enhance model utility.

Data sampling frequency and timing are a foremost concern for accurate CTS modeling. VID models that are fit to incomplete data sets can lead to inaccurate parameter estimation and poor reliability for clinical trial simulation. As with all infectious disease research, VID modeling benefits from increased sample size (28). A higher number of study participants facilitates reproducibility and generalizability of key data patterns, allows detailed examination of variability, and enhances model parameter identifiability (73, 74). Longitudinal sampling at key timepoints is also a prerequisite for accurate VID modeling. A common data gap for modeling viral infection is inadequate sampling during the early, often asymptomatic stages of infection when viral load expansion is best characterized (25). In these settings, household studies or longitudinal studies in high-risk groups often provide the best data for VID modeling (28, 29, 66, 75–79). Identifying the necessary frequency of sampling is also vital but varies across pathogens and stages of infection. For instance, for HSV-2 and EBV, sampling of study participants every 5 minutes, 2 hours, 6 hours, and 24 hours provided unique dynamical insights due to their rapid expansion and contraction phases (34, 80, 81). In contrast, for primary CMV in infants, weekly oral sampling was sufficient to capture the key features of shedding (29, 77, 78). Overall, VID models can be usefully applied to all human viral infections provided there is high-quality data for validation. For this reason, modelers should be involved in study design to ensure sampling captures key kinetic features.

## VID MODEL STRUCTURE

**Fig 4:**
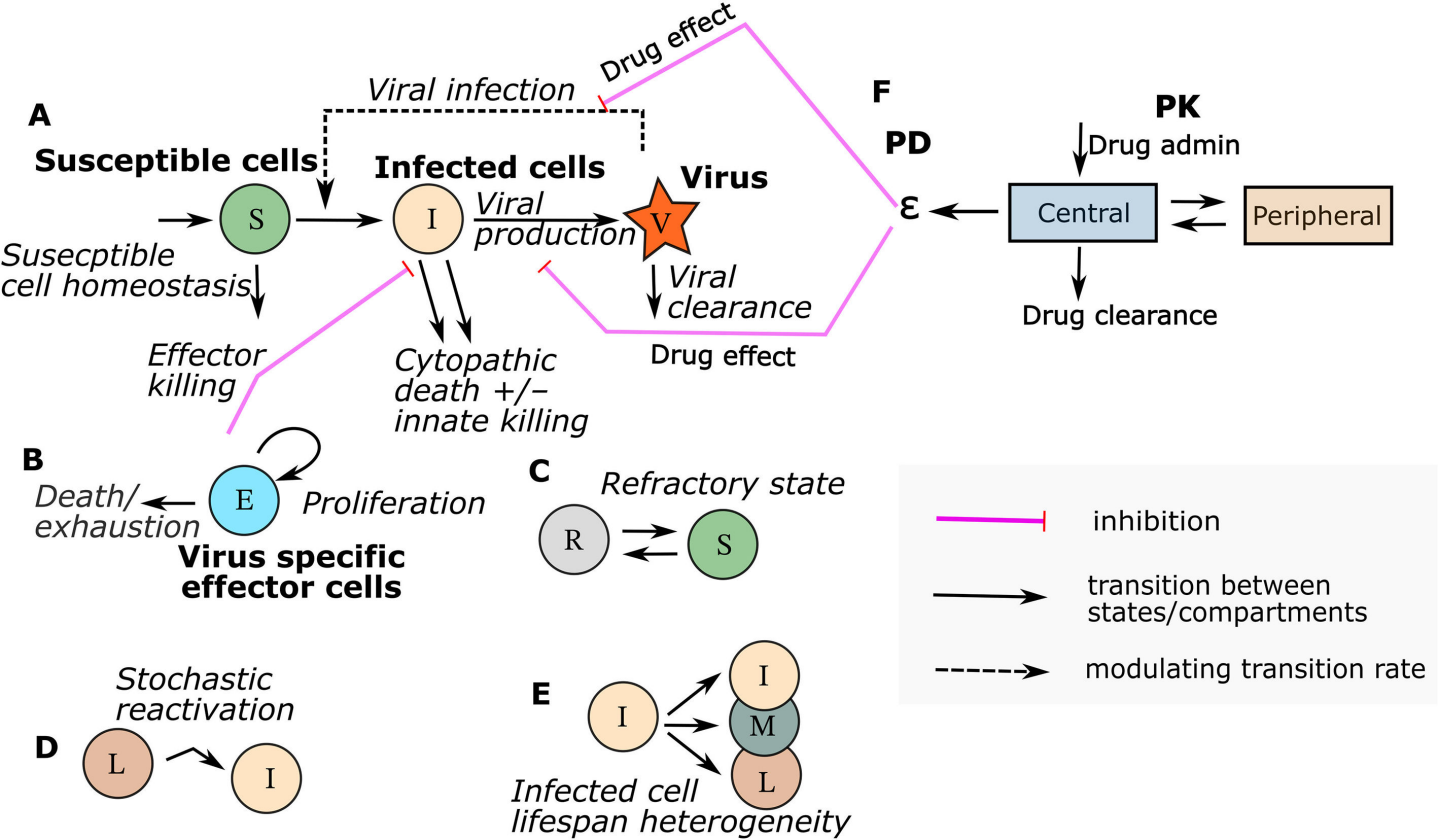
Basic components of CTS models with possible additional features catered to specific infection and data availability. (**A**) VID model demonstrating viral infection of susceptible cells, viral production by infected cells, viral clearance, and infected cell death due to viral lysis and effector cell killing. PKPD model integration with the VID model depends specifically on the drug mechanism. (**B**) Effector cells are assumed to proliferate, kill, and decay and may be divided into various subtypes depending on data availability. (**C**) Innate immunity may be modeled by susceptible cells becoming refractory at a rate proportional to infected cell number. Unlike effector cells, this component of immunity lacks memory and rapidly declines over time. (**D**) Herpes virus and HIV-1 models often include rates for reactivation from latency. (**E**) HIV-1 in particular is known to have various infected cell types with different productive lifespans. (**F**) PK and PD are layered onto the VID model according to mechanism of action.

The backbone of most VID models is a set of coupled ordinary differential equations (ODE) capturing rates of change of virus-susceptible target cells, infected cells, and viruses (6, 7, 13, 82). These three state variables are linked by several terms, including viral infectivity that captures the mass action effect of viral load proportionally converting target cells to infected cells; the death rate of infected cells; per cell production rates of viral genomes; and viral clearance rates (Fig. 4). Models are further specified to recapitulate unique features of a given viral pathogen. Examples of virus-specific model terms include rate of viral release from latency for human herpes viruses (30, 83); details of the multi-stage viral replication cycle for hepatitis B (5, 84–88); separate equations capturing the kinetics of cell-associated and cell-free viruses (89, 90); and time-dependent variability in infected cell lifespan for HIV and influenza (Fig. 4) (91, 92).

Immune response is often a vital addition to VID models and can be implemented in various ways corresponding to knowledge of the specific virus (Fig. 4). Innate immune responses lack memory and are typically assumed to be proportional in intensity to the concurrent number of infected cells, dissipating rapidly as infected cells decline (93, 94). Innate immunity has been implemented by tuning the conversion rate of target cells to a refractory state for respiratory viruses (28, 69, 95, 96), or by assuming increased cell death rates with intensifying infection (25). Other more detailed models consider different facets of innate immunity, including cytokine responses, phagocytosis, and antigen presentation; this level of detail will be necessary to capture mechanisms of host targeting antivirals (97).

Acquired immune responses are defined by memory with persistent effectiveness and slow contraction after pathogen clearance (Fig. 4). Another key feature of acquired immunity is polyfunctionality. In VID models, antibody responses may prevent cell entry (neutralization), clear free virus (binding), or lyse infected cells (antibody-dependent cellular cytotoxicity [ADCC]) (98, 99). T-cell responses may lyse infected cells, limit viral replication, or synergize with innate responses by protecting target cells from infection via cytokine signaling (63, 100). The amount of antigen presentation required to trigger a T-cell response is also often considered in models (101, 102). Some models of HIV include T-cell exhaustion, which is critical to long-term pathogenesis (103, 104).

A standardized and thorough literature review is a key first step in identifying the relative impact of different immune responses for a specific human virus. Testing VID models with competing immune response mechanistic assumptions for fit to observed virologic data can also be informative. While immunity drives the transition from viral expansion to clearance phases, models can sometimes also predict the timing, intensity, and phenotype of innate and acquired responses based on the more detailed contours of viral load kinetic curves, even in the absence of explicit immune data for model fitting (28). It is typically difficult to separate the specific impact of humoral and cellular responses absent longitudinal immune data, because of overlapping effects on infected cells. A new frontier in VID modeling is validating models against viral loads and concurrently gathered multi-omics immune data. (105, 106) While this level of detail is usually not required for robust antiviral CTS of virus-targeting drugs, these models will be helpful to project the effects of host-targeting immune agonists (107).

## VID MODEL FITTING AND SELECTION

As described elsewhere, a key methodologic development for VID, PK, PD, and joint antiviral CTS models is the application of population nonlinear mixed effects modeling (pNLME), which allows powerful parameter estimation using repeated longitudinal data (12, 108–110). Rapid fitting and model selection theory allows rigorous and transparent comparison of dozens of models with competing mechanistic assumptions (111). At present, comparative models with different equation structures are subjectively selected to test multiple hypotheses that may explain the observed data (14, 16, 25, 99). This is typically accomplished by removing or altering certain key terms. An area of potential growth for the field is the use of machine learning and artificial intelligence to less subjectively search the potential model space. Drawbacks of this approach are that it deviates from the traditional scientific method and that it may generate optimal models that include non-intuitive terms (such as polynomials), which are not readily translated to immunologic or therapeutic mechanisms.

Statistical models are used to assess fit to data, while also rewarding models for parsimony. Several software platforms, including Monolix, NONMEM, and packages in R, further assess parameter identifiability, correlations, and the need for covariates to best explain the data (112–115). After obtaining an accurate model, we believe that a further level of rigor should be obtained by formal assessment of non-fitted model variables, terms, and parameters to ensure realism (12). For instance, even if models are not fit directly to immune data, the magnitude of the immune response should be quantitatively compatible with experimental data. Similarly, models that result in massive target cell depletion beyond what is realistic clinically should be reconsidered. It is vital to perform a comprehensive literature review and consult with subject matter experts to ensure biological plausibility.

## VID MODELS FOR CHRONIC PERSISTENT INFECTIONS

Human viral infections fall into three broad categories: *chronic persistent, acute self-limited*, and *chronic recurrent* (Fig. 2). These distinctions are relevant as they fundamentally define clinical outcomes but also because they dictate proper selection of VID model type (Table 1). *Chronic persistent* viral infections, including HIV, hepatitis B, and hepatitis C, are characterized by infection stage-specific kinetics, which include a peak followed by a lower viral load “setpoint” (5, 116, 117). Plasma viral load steady state absent therapy is associated with a gradual progression toward deleterious clinical outcomes (49, 58, 118). Some viruses, including SARS-CoV-2, which are typically acute and self-resolved, can become chronic with persistent mucosal shedding patterns in severely immunocompromised individuals (119, 120).

**TABLE 1:** Data type and VID model selection according to type of viral infection

	Chronic persistent	Acute self-limited	Chronic recurrent
Viruses	HIV^*a*^^,*b*,*c*^ Hepatitis B^*a*^^,*b*^ Hepatitis C^*a*^^,*b*^ Persistent SARS-CoV-2 (rare outcome of infection in immunocompromised hosts)^*a*^	SARS-CoV-2^*a*^^,*b*^ Influenza^*a*^ RSV^*a*^ Monkeypox^*a*^^,*b*^ Dengue^*a*^ Zika^*a*^ Ebola^*a*^^,*b*^ Chikungunya^*a*^ CMV (primary) ^*a*^	HSV-2^*a*^^,*b*^ CMV (chronic)^*a*^ HHV-6^*a*^ HHV-8^*a*^ EBV^*a*^
Most common model type	Ordinary differential equations	Ordinary differential equations	Stochastic differential equations
Virologic kinetic model fitting features	1st, 2nd, and 3rd phase decay rate (based on fit to individual datapoints) Reservoir decay rate: HIV cure (based on fit to individual datapoints)	Viral load reduction at days 3 and 5 Viral clearance slope (based on fit to individual datapoints) Viral rebound (based on fit to individual datapoints)	Summary features: Shedding rate Episode rate Peak viral load Expansion slope Clearance slope Re-expansion frequency
Virologic trial endpoints	Plasma viral load suppression	Viral load reduction at days 3 and 5 Viral clearance slope (based on regression) Viral area under the curve	Shedding rate
Key unique model features	Therapy is often required for the VID model fits CTS modeling is sometimes possible without PK/PD models Variable host immune pressure	Therapy is not required for VID model fits CTS modeling is usually not possible without PK/PD models Variable host immune pressure	Fitting to individual trajectories impossible Inter-person variability captured by stochastic output and individual model parameters Variable host immune pressure Reactivation from latency

^*a*^ Infections with VID models.

^*b*^ Infections with CTS models.

^*c*^ HIV models include viral dynamic models to classify treatment and cellular models to classify cellular dynamics in the persistent HIV reservoir.

The original CTS models were designed for chronic persistent infections and have two distinguishing features. First, for models to infer key infection parameters of chronic persistent infections, it is necessary to perturb the steady state with treatment (5–7, 82, 117, 121). Effective antivirals typically induce bi- or tri-phasic viral decline (Fig. 2). A major goal of VID models is to infer the cellular and viral lifespans that cause these observed viral kinetic patterns and that are presumably the same before and during therapy. CTS modeling of viral kinetics *on* therapy is therefore required to infer viral-immune dynamics *off* therapy. Second, in contrast to acute self-limited and chronic recurrent infections, in many cases, it is possible to derive fundamental conclusions from VID models of chronic persistent infections without explicitly considering drug PK and PD. Key examples are listed in the following sections.

## HIV VID MODELS

The field of viral dynamics was initiated with the modeling of viral decay during early antiretroviral (ART) therapy for HIV (6, 7, 82, 122). Using a simple exponential decay model, Perelson and Ho revealed that the HIV RNA decay rate during treatment was a function of the lifespan of infected cells, representing an upper limit on the rate of observed decay for small molecular agents, which block various steps in the HIV replication cycle (6, 7, 82, 122). Viral load steady state preceding therapy was predicted to be far more dynamic than previously appreciated, with daily turnover of infected cells and replacement of virus; a second slower phase of viral decay corresponded with a longer-lived subset of infected cells, or with slower viral production rates (6, 7, 82) Along with the high error rate of the HIV reverse transcriptase enzyme, these discoveries necessitated concurrent use of multiple antivirals targeting different replication stages to avoid rapid selection of drug-resistant viruses (123). Subsequent more-detailed models assessed immunologic causes of variability in HIV viral load setpoint (124, 125), a critical determinant of time to AIDS which can vary over four orders of magnitude (Fig. 2). Models also demonstrated that some observed subtleties of viral decay timing and rate can be linked to the step of the HIV replication cycle inhibited by each class of drug (41, 126).

The original HIV model dramatically impacted drug development strategies. Thus, we consider it to be the first example of antiviral CTS. Remarkably, this model excluded critical features of infection, including immune responses. PK and PD were also not explicitly considered, and drug efficacy was reduced to a single constant parameter. This result highlights that the simplest models possible should always be favored for antiviral CTS (7, 122). More specifically, it demonstrates that antiviral CTS for chronic persistent infections does not always require PK equations, presumably because many drugs retain high potency even at drug trough, obviating the need for factoring in drug levels (127–130).

Vital PD models of highly active antiretroviral therapy (HAART) were developed to explain the extraordinary effectiveness of combination treatment. These studies demonstrated that certain agents, particularly protease inhibitors, achieve extremely high potency via cooperative target binding and that simultaneous use of drugs from different classes elicits multiplicative or synergistic effects to enhance potency and limit resistance (22, 23, 128–130). Remarkably, this groundbreaking work was performed after licensure trials and widespread use of HAART, but is relevant for treating other viruses.

With the advent of fully suppressive HAART, which extends survival by many decades, the focus of HIV VID modeling has shifted to a reservoir of persistent cells, which are the barrier to cure (131–138). A different set of VID ODE models focus on clonal behaviors of CD4+ T cells, including proliferation, death, and subset differentiation, rather than dynamics of HIV replication and spread (131, 139–141). Antiviral CTS models have proposed altering these processes, as well as activating latently infected cells followed by immunotherapeutic removal (14, 16, 132, 142–146). This change in model structure raises a more general concept for VID models, which is that their equations and assumptions must be adapted to the infection stage and therapeutic goals.

## HEPATITIS C VID MODELS

Mathematical modeling of hepatitis C (HCV) facilitated stepwise development of reliable short-course curative regimens, which are now the standard of care. Vital contributions included identifying viral decay patterns during treatment to infer turnover rates of virus and infected cells, identification of drug mechanism of action, delineating strategies to avoid drug resistance, dose optimization using PK approaches, and estimation of treatment duration required to achieve systemic elimination of virus. Like HIV, hepatitis C (HCV) has a pre-treatment steady state and exhibits triphasic decay during effective treatment (147). Initial modeling focused on interferon-alpha therapy, a systemic immune agonist that resulted in variable clinical responses with significant associated toxicity (148). This work highlighted the dose-dependent mechanism of action to be inhibition of HCV RNA production, though efficacy was limited by a 24-hour window of sub-potent activity (149–151).

Subsequent work examined HCV RNA decay patterns on various direct-acting antiviral treatments targeting the viral NS5B polymerase, NS3/4 protease, and NS5A viral assembly/release protein. These models identified that single HCV drugs are often polyfunctional. When added to interferon, ribavirin, which has minimal efficacy alone, increased sustained virologic response rates via dose-dependent inhibition of viral RNA polymerase as well as induction of mutational error catastrophe (116). These results provided a key template for how repurposed drugs can be combined to achieve meaningful clinical outcomes (152, 153). Nonstructural 5a (NS5A) inhibitors were predicted to potently inhibit both RNA synthesis and virion secretion to explain extremely short HCV RNA half-lives on this drug (154). Models also identified that non-nucleoside polymerase inhibitors and protease inhibitors may induce production of non-infectious, PCR-detectable HCV RNA, which accounts for the slow decline of HCV RNA in 6-week trials, which nevertheless achieved cure (155).

By synthesizing results with combinations of these drugs, modelers ultimately determined that first, second, and third phase HCV RNA decay reflect elimination rates of cell-free viral RNA, cell-associated RNA, and infected cells, respectively (57, 156, 157). As with HIV-1, apheresis experiments confirmed model-predicted rapid production and clearance of HCV RNA (158). Assessment of declining HCV RNA levels during the phase of surgical liver transplantation between recipient hepatectomy and donor graft implantation allowed precise confirmation of HCV RNA clearance rates absent viral production from hepatocytes (159). HCV RNA elimination rate was subsequently shown to be strain-specific based on varying stability of the replicase complex (160).

As with HIV, modeling identified a relatively low barrier to resistance with certain HCV antivirals due to the pre-treatment presence of all relevant single and double mutations resistance signatures (161, 162), necessitating dual or triple therapy in certain cases (163). Multiscale modeling, which included each step of viral RNA and transcription, predicted specific mechanisms of resistance for different drugs (164). Detailed modeling of the multi-stage viral replication process also suggested strategies for broadly targeting plus-strand RNA viruses, including coxsackievirus B3 and dengue (107).

While most HCV models did not consider drug levels, some modeling of pegylated interferon, polymerase inhibitors, and protease inhibitors in early development included PK equations and identified heterogeneous *in vivo* EC50 levels among trial participants (165–167). For interferon, these differences drove variability in treatment outcomes to a greater extent than variable drug levels across participants (148). PK parameters influenced both the rate of first and second phase decay under treatment (168). Leveraging concepts from HIV PD (22, 23, 128, 130), HCV single drug and combination regimens were also ranked according to potency (118).

The central achievement in hepatitis C treatment has been viral eradication of infected cells, leading to high rates of cure in treated patients, an outcome which was possible based on the absence of a latent reservoir. Modeling assisted in this process by using alanine aminotransferase (ALT) kinetics as a benchmark for infected cell death: ALT decline during direct-acting antiviral therapy suggested an infected cell half-life of 2.5 days (169). To determine the minimal duration of therapy required, multiscale models that accounted for links between the drug’s mechanism of action and viral decay rates. While current regimens range from 8 to 12 weeks depending on the degree of liver damage and viral genotype, modeling has suggested that even shorter regimens may be curative (170, 171).

## HEPATITIS B VID MODELS

Hepatitis B (HBV) is another chronic persistent virus. HBV dynamics were initially interrogated with partially effective direct-acting antivirals, which altered steady state to a variable degree, perhaps due to differences in individual drug potency (5, 172, 173). As therapeutic strategies matured, HBV DNA decay profiles typically followed a biphasic pattern (173, 174). Like HIV, the addition of a second agent did not dramatically change viral clearance rates (174), though there was additional benefit in avoiding selection of resistance in certain cases. Differential viral clearance rates were noted according to initial viral load, as well as presence of the eAg (e antigen) in plasma, suggesting that immune responses partially determine the death rate of infected liver cells (175, 176).

Detailed assessments of primary infection prior to steady state also assisted with parameter estimation (177, 178). An important feature of VID models for HBV was identifying the determinants of HBV spontaneous elimination, which occurs in ~90% of acutely infected people, despite initial infection of nearly every hepatocyte. High rates of refractory cell production, sufficient cellular and humoral immune responses, and infected cell proliferation were predicted to be necessary for achieving spontaneous cure versus persistence (87, 179–181). A low viral inoculum dose may counterintuitively limit the effectiveness of subsequent immune responses (182). Overall, these studies suggest that observed viral decay patterns during treatment occur due to drug and host immune effects.

Initial trials were performed with the immune agonist interferon, which incompletely blocks viral production and facilitates infected cell elimination, as well as directing acting small molecular agents which only target replication. Like HIV, prolonged treatment dramatically reduces HBV DNA loads in a biphasic manner but usually does not result in a cure. Models attributed biphasic decay to two populations of infected cells, which differ according to HBV transcriptional activity (183). HBV RNA is also being assessed as a possible infection biomarker: recent modeling under therapy targeting nucleocapsid assembly showed much more rapid HBV RNA clearance relative to HBV DNA, reflecting clearance of pre-formed versus fully formed particles during the first few days of treatment (86).

Unlike HIV and hepatitis C, hepatitis B is not usually lytic to its host cells. Accordingly, models estimate considerably longer estimates of infected cell lifespan (86). A potential strategy is therefore to augment endogenous immune responses. More rapid clearance of hepatitis B DNA during interferon treatment relative to direct-acting antiviral agents suggests that transcriptionally active and inactive infected cells become more susceptible to elimination by immune cells under treatment (183). Modeling of HBV targeting neutralizing antibodies showed that these products may facilitate the elimination of HBV DNA and Hep B surface antigen (sAg) while also blocking cellular release of the virus (184).

A key element of more recent HBV modeling is the exploration for markers of functional cure. Unlike HIV, hepatitis B’s reservoir consists of variable numbers of extrachromosomal cccDNA molecules, which are part of the replication cycle (185). Because cccDNA cannot be measured in plasma in humans, longitudinal measures are impossible in trials. Recent humanized mouse models suggest that nearly all potential biomarkers, including HBV DNA, HBsAg, HBeAg, and HBCrAg, have long-term decay profiles in general accordance with cccDNA levels (85, 186). HBV models, therefore, often incorporate detailed steps of the viral replication cycle to predict forces driving cccDNA levels, including cell proliferation, cccDNA amplification, and cccDNA degradation (88, 187). Elegant multi-scale models demonstrated that observed HBV decay kinetics can be related to different drugs targeting sequential steps in the viral replication cycle (188). For instance, RNA interference compounds showed blockage of HBV DNA, HBsAg, and HBeAg relative to direct-acting antivirals, which only target HBV production (189). Studies using a nucleoside polymer in addition to tenofovir and pegylated interferon suggested that monophasic HepBsAg decline and HBV reduction to below detection limit are possible surrogate outcomes for functional cure (190, 191). Each of these concepts is being leveraged for antiviral CTS approaches to HBV cure. As strategies evolve, it is likely that more granular PK and PD models will be useful to accurately simulate outcomes.

Finally, VID models are increasingly considering the importance of hepatitis D (HDV) co-infection. HDV requires prior hepatitis B infection to infect the human liver but can then accelerate progression to cirrhosis and liver cancer. Modeling has also established HDV clearance kinetics during interferon therapy targeting both viruses (192, 193) and HDV nucleoside analogs (194).

## VID MODELS FOR ACUTE SELF-LIMITED INFECTIONS

*Acute self-limited* infections are eliminated from the body even in the absence of treatment and are defined by successive viral expansion and clearance phases (Fig. 2, Table 1). Examples include respiratory viruses such as influenza and SARS-CoV-2, which have been extensively and accurately modeled based on sampling of oral and nasopharyngeal passages for viral load (4, 28, 69, 70, 95, 96, 195). Full CTS models have been applied to SARS-CoV-2 trial data as described below, but have yet to be fit to influenza viral load data from multiple trials showing clinical benefit with oseltamivir and baloxavir (196, 197). Monkeypox is a self-resolving genital infection that was successfully modeled during recent outbreaks with fits to viral loads and lesion size (198–202). Other examples of systemic acute self-limited infections with VID models are Ebola (203, 204), dengue (205–208), and Chikungunya virus (209, 210). Details of the full CTS application to several of these viruses are described below.

Deterministic ODE models are well-suited for fitting to highly diverse individual viral trajectories from untreated people with acute viral infections (12, 13). SARS-CoV-2 viral load trajectories vary from low peak viral load with rapid elimination (Fig. 2) to prolonged infection over months in immunocompromised individuals (211–213), and may differ according to a person’s vaccination status, age, and variant of concern (28). To account for this variability, each successfully modeled individual is assigned a specific parameter set. VID models can often distinguish different viral load patterns according to the effectiveness of innate and acquired immune responses (28). With the development of a full antiviral CTS model, it is then possible to analyze whether certain viral load profiles are associated with better or worse treatment outcomes (24).

A major challenge with VID models of acute self-limited infections is uncertainty in the number of target cells, particularly for systemic viruses. These estimations are complicated by multiple potential sites of replication, particularly in the upper airway. Similarly, most models overlook the likely high variability in viral inoculum dose across infected individuals, as this information is only measured and controlled in human challenge trials (214, 215). The modeling community appropriately seeks uniformity in estimates for these model starting conditions, but this unfortunately does not guarantee their accuracy. Another challenge is delineating the best approach to model immune responses. The presence of bi- or triphasic decay in SARS-CoV-2 data, as well as the presence of a viral load plateau for influenza rather than a sharp peak, often necessitates the incorporation of innate and acquired responses to optimize fit. It is important to test competing models with and without these responses (28, 96).

## VID MODELS FOR CHRONIC RECURRENT VIRUSES

Human herpes viruses are *chronic recurrent* with unpredictable episodic mucosal shedding patterns (Fig. 2, Table 1) (216–219). Individual shedding episodes often have features resembling acute self-limited infections. Therefore, deterministic ordinary differential equation models may be capable of reproducing individual episode kinetics. However, for HSV-1 and 2 (220–222), EBV (30), and HHV8 (31, 223), each episode may be the sum of dozens of infection micro-environments that concurrently contribute to the virus, which often leads to multi-peak episodes with non-monotonic expansion and clearance phases (224). This type of data is not amenable to deterministic model fitting. In addition, the timing of episodes within an individual is impossible to predict (224). For these reasons, stochastic models that include multiple micro-regions that may or may not be linked by viral seeding or immune cell trafficking are the preferred VID model type for this type of data.

Stochastic ordinary differential VID models generate unique viral load trajectories with every simulation, even if model structures and parameter values are held constant. Therefore, fitting criteria for these models are not based on reproducing individual viral load trajectories, but rather on summary statistics of the observed data which can be characterized in detail including quantitative shedding rates (percentage of time shedding at different viral load strata); episode rate; episode duration; first, peak, and last positive viral loads in episodes; and viral expansion and clearance slopes (89). These values vary dramatically across episodes. Other data features may be extracted with machine learning or clustering algorithm techniques (199). Approximate Bayesian Computation (ABC) provides a natural framework for parameter estimation in this context (30, 225). We previously fit models to frequency distributions for summary statistics and used rejection sampling to derive distributions of acceptable parameters (26, 27, 34, 220). A critical detail for this type of VID model is that simulated sampling intervals and durations match those in the study cohort (27).

The nature of data fitting is particular to the herpes virus. For HSV-2, the same model and parameter values surprisingly generate the variable distribution of observed shedding rates among individuals in clinical trial placebo arms. All variation in model output is due to the limited sampling duration in the trials (30–60 days) and the stochastic output of the model. For CTS, it is therefore unnecessary to create “digital twins” who match trial participants according to shedding characteristics (27). On the other hand, for EBV and HHV8, observed shedding features are extremely sensitive to input parameters, even if sampling only occurs over 30 days (30, 31, 223). To create the full variability of shedding that would be observed in the placebo arm of a trial, a wide range of data-informed parameter inputs is necessary.

## SMALL MOLECULE PK MODELS

Phase 1 clinical trials are conducted in healthy and/or infected humans primarily to assess drug safety but also to provide longitudinal drug levels to inform design of PK models (Fig. 1 and 3). PK models intend to capture drug levels over time. Equations consider drug absorption, distribution, and clearance (114, 226). Some PK equation sets also capture metabolism of antiviral drugs from their prodrug state to active form (25). In most cases, only plasma drug levels are available from humans, but models can be trained to reproduce tissue drug levels if data are available. PK modeling has matured considerably with the advent of non-linear mixed-effects approaches, which allow subtle permutations of model structure to achieve better data fits (227). For translatability to human infection, it may or may not be necessary to account for covariates that may impact drug clearance, such as age, gender, renal function, hepatic function, or infection severity (228).

As PK models reflect constant changes in drug level and PD models reflect instantaneous potency, combining PK and PD models is a common approach to estimate concentration-dependent drug potency over time (Fig. 1) (26). Potency can then be averaged to estimate a single crucial parameter, the mean drug potency during dosing, which varies from 0% to 100% (229). Unfortunately, because this approach requires inputting the *in vitro* EC 50, which is not predictive of human efficacy, initial estimates of potency are frequently inaccurate. Nevertheless, certain key principles can be carried forward to human studies, such as the need for a loading dose for drugs with a long half-life, as well as the need for multi-dose phase 2 studies, given the uncertainty of *in vitro* EC50 values for initial dose prediction (27).

## NEUTRALIZING ANTIBODY PK MODELS

Monoclonal neutralizing antibodies are being engineered to prevent viral entry into cells. Monoclonal antibodies usually have favorably long half-lives and are specifically designed for high potency (61, 230–232). Traditional PK models are well-suited for capturing serial antibody levels following intravenous or intramuscular dosing (17, 233). Similar concepts and modeling approaches can be applied to synthetic large-molecule antibody mimetics such as eCD4Ig for HIV (14).

More complex PK models are needed to probe novel delivery systems, including viral vectorized delivery of a transgene encoding antibody production (14, 15, 234). These systems use non-pathogenic viruses delivered to muscle and liver cells, where constitutive expression of the antibody may occur. This approach has the potential to provide constant antibody levels over years but is challenged by anti-vector and anti-drug antibody responses, which accelerate elimination of antibodies from plasma (14). Variables and equations to capture these phenomena are necessary to optimize model fit to data.

For monoclonal antibodies, integration of PK models with VID and PD models has considerations beyond the modeling of small molecular therapies. Antibodies may have binding capabilities (98, 235), leading to more rapid viral clearance, or ADCC, leading to more rapid elimination of infected cells (236). Therefore, models, which include and exclude terms that capture these mechanisms, can be compared for data fit and parsimony. For HIV, there is increasing evidence that binding of monoclonal antibodies to free virus promotes immune complex formation, which, in turn, can generate virus-specific CD8+ T-cell responses (237, 238). This potential vaccinal mechanism can be formally assessed with comparative model testing. Finally, it appears that HIV viremia, as well as the presence of antivirals, may accelerate clearance of therapeutic monoclonal antibodies, a finding which might be explained with advanced PK models in which the interaction between virus and drug is bidirectional (239). Finally, some preliminary work suggests that plasma monoclonal antibody concentrations needed for prevention may differ from concentrations needed for treatment (17).

## CELLULAR IMMUNOTHERAPY PK MODELS

Borrowing from cancer therapeutics, autologous and third-party immune cellular immune therapies are being developed for persistent viral infections, including for CMV, BK virus, and adenovirus after stem cell transplantation (240–242), as well as for HIV and HBV cure (243, 244). Development of PK models for these interventions is in its infancy but will borrow heavily from modeling of CAR T-cell therapy for cancers (245), as well as the immune equation portion of existing VID models. Cellular therapies are living treatments designed to have memory and therefore re-expand and differentiate toward effector subsets with re-exposure to viral antigen following reactivation or therapeutic vaccination. Therapeutic effects may synergize or compete with endogenous immune responses, which are dynamic in immunocompromised hosts, depending on shifting immunosuppressive regimens (246). Repeated antigen exposure may induce exhaustion (243). Modern PK models will need to consider these complex behaviors and will borrow from existing VID models.

## VECTOR DELIVERY OF GENE EDITING ENZYME PK MODELS

As described above, vectorized delivery of genetic payloads is a possible way for therapeutics to access infected cells. This method is being used for various types of DNA cleavage enzymes to target latent viral genomes, including HIV, HSV, and hepatitis B (247, 248). PK models, which have been developed for this approach, must account for the kinetics of viral vector delivery, gene expression, and anti-vector antibody responses, with attached PD models accounting for the efficiency of enzymatic cleavage and terminal mutagenesis of viruses (14, 15, 249).

## DEFECTIVE INTERFERING PARTICLE PK MODELS

Another strategy being developed for the cure of HIV and other chronic viruses is co-infection with defective interfering particles (DIP), which are designed to outcompete natural infection (250). A related idea is viral gene drive in which a non-pathogenic virus is introduced with the goal of supplanting the wild-type infection (251). Modeling is integral to these strategies. Proposed PK models resemble a parallel VID model in which the therapeutic DIP lacks infectivity. Ideally, models are trained to pathogen and DIP viral loads (251).

## PD MODELS

Pre-clinical drug development starts with screening a large library of existing or synthetic compounds for concentration-dependent direct antiviral activity and then applying PD models to this data to quantify potency (Fig. 1 and 3). The translational relevance of drug screening is determined by careful selection of assay conditions. During the early months of the COVID-19 pandemic, drug screening with physiologically irrelevant cell lines resulted in pre-selection of repurposed drugs with low potential for antiviral activity in humans, as demonstrated with subsequent experiments in more relevant human respiratory epithelial cell lines (252, 253). Standardized and reproducible readouts of antiviral replication are also crucial to incorporate. For HIV drug development, single-cycle assays have been developed to ensure that a drug’s impact is not overestimated due to experimental inhibition of multiple rounds of infections (22, 23, 126, 128, 129). Organ on a chip or *ex vivo* organelle approaches are emerging technologies that might provide more accurate translation of drug potency to humans.

PD modeling is characterized by fitting a single equation to static pre-clinical *in vitro* dose-response data (Fig. 1) (254). Model parameters include the drug concentration at which infection is inhibited by 50% (*in vitro EC50*), dose-response slope (Hill coefficient), and maximum drug effect (Emax). Given the limited parameter space and high reproducibility of the data, PD models are highly identifiable and reliable and allow detailed specification of drug potency *in vitro* (152). Antiviral activity at nanomolar concentrations is often considered a pre-requisite for further antiviral testing.

A critical discovery was the high variability of the dose-response slope according to the drug mechanism of action. An elegant assessment of all available HIV small-molecular agents demonstrated that converting the x-axis (drug concentration) and y-axis (drug effect) to logarithmic forms allows identification of this dose-response slope (22, 23, 128–130). Certain drugs, such as HIV nucleoside analogs, have a slope of approximately one, allowing the possibility of intermediate drug efficacy at a wide range of concentrations. HIV protease inhibitors have a very steep dose response (slopes 3–5), denoting a very narrow drug concentration separating absent from complete effects (23). The mechanism underlying the steep dose response is cooperative drug binding to its target (130). The differences between low- and high-dose-response slope have been compared to a dimmer switch versus an on-off switch and, along with drug PK characteristics, are important to consider for optimal dosing. Summary metrics such as the instantaneous inhibitory potential (IIP) capture both EC50 and hill slope effects on potency (23, 233).

The central limitation of PD modeling is that *in vitro* assays do not recapitulate *in vivo* conditions which may differ according to multiple variables, including drug delivery to cells, protein binding, different metabolic cell states, and parameters of blood flow. To address this issue, the PD portion of antiviral CTS models can be solved for the *in vivo EC50*, defined as the plasma concentration required to inhibit viral infection by 50% (26, 27). The *in vivo* EC50 often differs significantly from prior *in vitro* estimates (Table 2). Its estimation is one of the clearest benefits of antiviral CTS models.

**TABLE 2:** Examples of *in vivo* IC50 estimates from CTS modeling

Drug	Virus	Potency adjustment factor (*in vivo* IC50/*in vitro* IC50)
Nirematrelvir/ritonavir	SARS-CoV-2	37 (omicron variant) 61 (pre-omicron) (24)
Molnupiravir	SARS-CoV-2	0.13 (omicron variant) 2.64 (pre-omicron) (42)
Bamlanivimab	SARS-CoV-2	~2,000 (pre-omicron IC90) (255)
Etesevimab	SARS-CoV-2	~6,000 (pre-omicron IC90) (255)
VRC01	HIV-1	630 (22)
Acyclovir	HSV-2	1 (26)
Famciclovir	HSV-2	1.7 (26)
Pritelivir	HSV-2	7 (27)

## DRUG COMBINATION PD MODELS

Groundbreaking work on HIV drug combinations provided a detailed mathematical framework to assess how potency increases with two or more drugs (22, 23). Drug combinations can range in order of desirability from antagonistic (drug B lessens potency of drug A or vice versa) to additive (drug A and B have overlapping activity such that only the limited non-overlapping activity of drug B adds to overall potency) to independent multiplicative (drug B works independently with non-overlapping activity to drug A) to synergistic (potency exceeding that predicted by multiplicative models). Generally speaking, drugs with equivalent mechanisms are more likely to be additive, whereas those working at sequential steps in the viral replication cycle are more likely to be multiplicative or synergistic (128). For HIV, the latter combinations are also more likely to avoid drug resistance, as different mutations are required to bypass different antiviral families (22).

More recent work with experimental readouts incorporating a matrix in which concentrations of both drugs are varied suggests that drug combinations are not exclusively additive, multiplicative, or synergistic (152). Rather, the nature of the effect is dependent on the concentration of both drugs. Updated models account for this possibility and are important when combined with PK models because concentrations of both drugs vary over time (254). A subtle feature of these models is that the potency benefit provided by synergistic dual mechanism combinations is most pronounced when the potency of a single agent is incomplete. It may be most important to leverage synergy when the individual drugs are not sufficiently potent on their own or cannot be dosed to achieve full potency (203). This concept might be leveraged with the use of repurposed drugs, as part of a rapid pandemic response.

## FULL CTS MODELS

Full antiviral CTS models involve merging data-validated VID, PK, and PD models and fitting them to viral load data from the treatment arm of clinical trials (Fig. 1 and 3) (26). The integration of these models into a single equation set allows stepwise comparative mechanistic model testing and parameter identification (24). In addition, modeling drug levels and activity, concurrent with antiviral immune responses, ensures that all drivers of observed viral kinetics are considered. VID models that ignore PK and PD and reduce antiviral efficacy to a single parameter overlook the fact that overall drug efficacy is a synthesis of drug half-life, dose, and dosing intervals. In isolation, PK and PD models neglect that elimination of virally infected cells occurs due not only to therapeutic effects but also mounting, non-linear innate and acquired immune responses which differ in timing and intensity by pathogen (24).

Fully integrated CTS models fit to viral load data from human trials accurately estimate the concentration of drug required in plasma to limit infection (24, 27, 42). Therefore, CTS models are superior to *in vitro* PD models for optimal dose selection, timing of treatment initiation, duration of therapy, strategies to avoid resistance, and virologic endpoint selection. Importantly, fully synthesized CTS models are possible with every type of VID, PK, and PD model described above.

## MODELING DIFFERENT MECHANISMS OF ACTION IN ANTIVIRAL CTS

Integration of VID, PK, and PD models must accurately capture the drug mechanism of action. Neutralizing antibodies and small molecular entry inhibitors decrease the value of the term denoting infection of new cells in a dose-dependent manner (17, 233, 256). Polymerase inhibitors and protease inhibitors are assumed to lower viral replication rate in cells, though a more detailed approximation of the replication cycle can be modeled in which these mechanisms are considered separately (6, 257). HIV chromosomal integrase inhibitors work before viral RNA replication and lead to slightly earlier viral decay than protease inhibitors, which are associated with a brief lag before viral decline (41, 126). Mutagenic agents such as ribavirin and molnupiravir convert viral genomes to a PCR detectable but non-infectious genome, which necessitates a separate model variable (32, 42). Oseltamivir prevents influenza egress from infected cells, requiring a different set of equations to capture this effect (197). Multiple interventions may accelerate the death rate of infected cells, including interferons and CAR T cells (58, 116, 258).

Some repurposed drugs have unknown mechanisms of action but reduce viral load (259). In this case, various competing models assuming different mechanisms can be assessed for fit to the data. Similarly, neutralizing antibodies may have adjunctive mechanisms such as ADCC, leading to more rapid cell death or binding leading to more viral clearance (236). Models with and without these mechanisms can be tested to assess each mechanism.

## DATA NEEDS FOR INTEGRATED ANTIVIRAL CTS MODELS

A critical early determination is extracting features of virus-specific data that are most appropriate for model fitting. In the case of chronic, persistent, and acute self-resolved viruses, fits to individualized data should be prioritized. This approach is powerful because it may identify host, viral, or drug features that determine treatment effectiveness (24). It is also crucial to fit to established virologic surrogate endpoints to ensure key trial outcomes are reproduced. Model output can try to recapitulate other important data features, such as virologic rebound (24) or selection of drug-resistant variants (260). Often, a key step is model calibration according to the specific study populations in trials, which may vary in critical ways according to age, degree of immunosuppression, timing of intervention, and viral variant.

Antiviral CTS often relies on data from multiple sources. For instance, VID models may first be optimized based off of a natural history cohort, and PK models may be informed by phase 1 trial data, whereas the CTS model is validated against phase 2 or 3 trial output (Fig. 3). While this data integration from multiple studies is typically necessary, optimal modeling data would include drug level, viral load, and viral sensitivity data from the same trial participants, a standard which has been met for HIV broadly neutralizing antibody studies (261, 262). Phase 1 and 2 studies that include multiple doses and a dose-response relationship with virologic output also enhance model validation and assist in guiding accurate predictions for dose optimization (27, 263, 264). Because intense sampling is only possible in early-stage trials, we advocate for close integration of modeling teams with drug developers during this period.

## PARAMETER ESTIMATION FOR ANTIVIRAL CTS MODELS

It is important for modelers to carefully decide which parameters should be fit when conducting antiviral CTS. Generally speaking, if VID parameters pertaining to viral replication and immune response vary across placebo recipients, then these are likely to be sources of viral kinetic variability in those receiving study drugs. PK parameters can only be individualized if serial drug-level data are available along with viral loads. Otherwise, a less optimal but common compromise is to input fixed population mean values to characterize changes in drug levels over time (24, 42). Most antiviral CTS models assume that all PD parameters derived *in vitro* are applicable to the fully integrated antiviral CTS model, other than the *in vivo* EC50. A crucial feature of CTS is that previously derived realistic ranges of nearly all VID, PK, and PD parameters can be imputed, limiting issues of parameter identifiability despite the increasing complexity inherent to integrating three model types. Nevertheless, it is rare that all PK, PD, and VID parameters are fully identifiable for all trial participants, and this must be acknowledged as a limitation in published models (12, 73, 74, 265).

A common tension in the field is to choose between simple models that favor structural identifiability and more complex but realistic models for which most parameter values remain uncertain. Analytical and numerical approaches have been developed to address this issue, which is also mitigated by fitting models to detailed, longitudinal data. Nevertheless, it is critical to admit parameter value uncertainty and to test whether this uncertainty impacts CTS model predictions (12, 73, 74, 265, 266).

## THE *IN VIVO* EC50 FROM ANTIVIRAL CTS MODELS

If antiviral CTS models assume values of the *in vitro* EC50, then fitting to the treatment arm virologic data is often poor (27). Alternatively, the effect of the drug may be forced on other non-treatment-related model parameters, resulting in unrealistic differences in parameter distributions between control and treatment arms. It is therefore important to solve the antiviral CTS model for the *in vivo* EC50 and to check whether the VID model parameters are equivalent between placebo and treatment. The *in vivo* EC50 can be tuned to account for all drug mechanisms.

In most published examples, model output is very sensitive to even twofold changes in the *in vivo* EC50, which can significantly improve or diminish model fit to data. For instance, slightly lowering the *in vivo* EC50 in simulations leads to increased rates of viral clearance in deterministic models applied to acute viral infections (SARS-CoV-2) and reductions in viral shedding rate for stochastic shedding models (HSV-2) (24, 27). The *in vivo* EC50 can be a highly relevant number for drug developers and regulatory agencies. By providing a precise target that drug trough levels should exceed, it vastly improves on the more widely used benchmarking approach, which assesses whether peak and trough concentrations exceed the *in vitro* IC90. Because the *in vivo* EC50 is independent of dose and duration of infection, it is central for projecting outcomes of other dosing strategies (24, 27). It is also possible to make conservative estimates of viral load trajectories prior to *in* vivo EC50 estimation, as has been done for Mpox and Ebola (200, 203). Overall, solving the *in vivo* EC50 should be a key priority for CTS models.

We defined *the potency adjustment factor* as the ratio of *in vivo* EC50 to *in vitro* EC50 to estimate whether *in vitro* estimates are reliable for predicting clinical trial outcomes (Table 2). The potency adjustment factor is widely variable across drug-virus pairings with a trend toward *in vitro* estimates overestimating potency within an infected person. In particular, monoclonal neutralizing antibodies targeting HIV and SARS-CoV-2 appear to be far less potent in people than *in vitro* (17, 255, 256), perhaps because antibodies must achieve higher concentrations *in* vivo to stop cell-to-cell spread. Conditions underlying highly variable potency adjustment factors for small molecules cannot be determined by CTS models but may include differences in delivery, cellular metabolism, or unmeasured protein binding or drug sequestration in uninfected cells. PK models that capture plasma and intracellular drug levels over time in a person may be able to identify infected tissue drug levels required for efficacy (25). Unfortunately, these data are rarely available. The *in vivo* EC50 is intended to be convenient because obtaining plasma drug levels is routine and does not require serial biopsies.

## OPTIMIZING TIMING OF THERAPY TO LEVERAGE CONCURRENT IMMUNE RESPONSES ANTIVIRAL CTS MODELS

Fully integrated antiviral CTS models capture how antiviral therapies are impacted by ongoing immune responses, and, in turn, how treatment sometimes indirectly blunts immune responses (25). These bidirectional interactions are critical to accurately predict trial outcomes. In most people, respiratory viruses like SARS-CoV-2 and influenza are ultimately eliminated by local immune responses and follow somewhat similar kinetic patterns, though pre-existing host immunity has a profound impact on the amount and duration of viral shedding (119, 267). During chronic, systemic infections such as hepatitis B and HIV, immune responses are insufficient to eliminate viral replication but dictate the extent of viremia, which, in turn, predicts the rate of disease progression (268–270). For most acute infections, immune responses intensify over time, leading to synergy with antiviral agents, but this may occur over a period of hours (HSV-2) (89), days (respiratory viruses) (99), or months (HIV) (271), highlighting the need for virus-specific VID models.

Clinical trial simulations of SARS-CoV-2 treatment demonstrate the crucial nature of therapeutic timing relative to innate and acquired immune responses. Early during the SARS-CoV-2 pandemic, multiple modeling groups predicted that antiviral therapy would have limited efficacy in hospitalized individuals (13, 25, 35, 36, 95) because most morbidity is related to maladaptive immune responses rather than viral replication during this late stage of disease (272). Subsequent advocacy for earlier treatment was validated in outpatient early treatment trials of nirmatrelvir/ritonavir (43), molnupiravir (44), and remdesivir (45), as well as several viral spike protein-targeting bNAbs (231, 273, 274), all of which demonstrated significant relative reductions in hospitalization and death when given within 5 days of symptom onset. Importantly, post hoc analysis demonstrated that extent of viral reduction is a valid surrogate outcome for reduction in hospitalization (60, 275).

A subsequent puzzle emerged when nirmatrelvir/ritonavir (which showed a 90% reduction in hospitalization and death in the EPIC-HR trial, leading to immediate emergency authorization by the FDA) was associated with high rates of viral and symptomatic rebound in community-based cohorts, despite no signal of rebound relative to placebo in EPIC-HR (43). The perception of frequent viral rebound among patients and providers dramatically limited the use of nirmatrelvir/ritonavir in high-risk patients, which regrettably likely resulted in ~30000 deaths in the United States (276). Another surprise was that nirmatrelvir/ritonavir failed as post-exposure prophylaxis despite its high antiviral potency (277).

**Fig 5:**
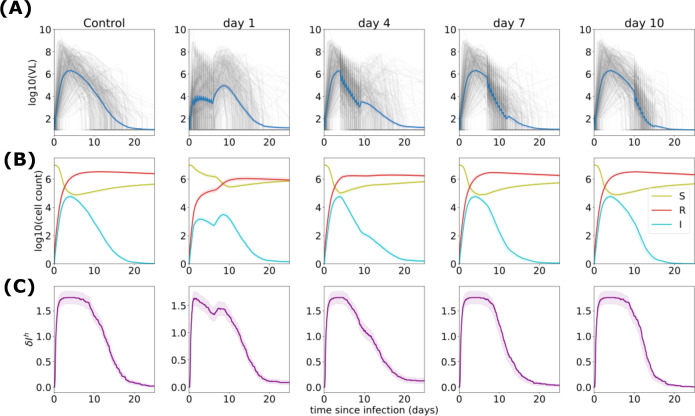
A mechanistic explanation of viral rebound following nirmatrelvir/ritonavir treatment**.** Earlier treatment is associated with greater preservation of susceptible cells, lack of elimination of infected cells, and blunting of innate immune responses, all of which allow viral rebound when treatment is completed after 5 days. Columns denote timing of treatment initiation. Treatment at day 1 of infection presents the highest risk of rebound, while day 4 treatment has a moderate risk. (**A**) Gray lines are individual simulations, and the blue line is the median output. (**B**) The middle row shows median projections for susceptible cells (S), infected cells (I), and innate immune-induced refractory cells (R). (**C**) The bottom row denotes the overall force of the innate immune response over time as projected by the data-validated CTS model (24).

To address these discrepancies, we fit a CTS model to virologic trial endpoint data from EPIC-HR and PLATCOV, as well as individualized longitudinal viral load data in treated and untreated individuals in PLATCOV (24). The validated models made the counterintuitive conclusion that the increased rate of rebound in community studies was because therapy was typically given within the first day of symptoms rather than several days after symptoms as in EPIC-HR. Earlier therapy given for 5 days was predicted to not eliminate all infected cells or viruses, preserve a greater proportion of target cells, and blunt mounting innate immune response (24). When therapy is stopped, conditions are suitable for infection to recrudesce (Fig. 5). This framework was extended to explain the failure of nirmatrelvir/ritonavir as post-exposure prophylaxis, which occurs even earlier before symptoms are evident (24). This and another complementary modeling study suggested that extending therapy by several days would eliminate rebound, as this provides sufficient time for an adaptive immune response to remove remaining infected cells (278). This hypothesis is supported by therapeutic trials studying monoclonal antibodies with longer half-lives in which rebound was not observed and in efficacious post-exposure prophylaxis trials with these same antibodies (60, 231, 273, 274, 279), and has since been validated in a trial of treatment for severely immunocompromised individuals (280).

Accurate clinical trial simulation of herpes simplex virus-2 (HSV-2) presents a different scenario because of intense and rapid tissue-localized immune responses during genital HSV-2 reactivations that initiate within 12 hours of viral detection and predominate several hours later, leading to a rapid viral clearance (34, 38, 281). Antiviral treatments dosed after immune responses dominate locally have no added benefit within a single infection micro-environment/genital ulcer, though therapy does prevent seeding of new spatially discrete regions, which may shorten the duration of a reactivation (26, 281). The rapidity of HSV-2 expansion and clearance kinetics explains why thymidine kinase inhibitors (acyclovir, famciclovir, and valacyclovir) with short half-lives (~3 hours) must be therapeutically dosed immediately after symptoms develop and why breakthrough shedding persists even if these agents are given at maximal doses twice daily (26, 281). Modeling predicted the need for long-half-life agents to overcome these barriers and was validated with close recapitulation of shedding outcomes in a multi-dose phase 2 trial of pritelivir, a helicase primase inhibitor with a half-life of ~70 hours (27).

HIV-1 infection presents an important counterpoint to these examples. While it is recognized that host CD8+ T-cell responses are a key determinant of viral load setpoint (125, 270), the virologic treatment response to effective antivirals is nearly equivalent across individuals, assuming selection of an appropriate regimen (282). Given a drug-sensitive virus and drug adherence, therapeutic response is nearly universal (283). Viral clearance rates differ little among individuals regardless of plasma viral load. Subtle differences are more likely to be related to the drug’s mechanism of action than host immunity (126).

Infections such as cytomegalovirus (CMV) take on a wide diversity of viral kinetic and disease profiles according to the degree of immunodeficiency in the infected host (284). Some individuals clear their infection spontaneously, while others require antiviral treatment (240, 285). Less commonly, patients with severe immunosuppression do not respond sufficiently to existing antiviral therapy and either succumb to infection or develop drug resistance and prolonged infection (286, 287). Overall, these results suggest that the efficacy of antiviral therapy hinges on some co-existing antiviral immune pressure (59, 284). This concept extends to multiple other viral infections in these immunocompromised populations, including adenovirus, BK virus, and respiratory viruses (240, 285). As therapies become available, these heterogeneous viral load kinetics will need to be captured accurately by CTS models.

## DOSE OPTIMIZATION WITH ANTIVIRAL CTS MODELS

Once an antiviral CTS model is validated against viral load data for one or more doses of a drug, it is possible to simulate other trials that vary according to key features. The dose may be changed to predict whether this will lead to more favorable virologic outcomes (27). Increasing the dose is often associated with a more rapid elimination of the virus, but whether this is possible is specific to the virus. For instance, the upper limit of HIV clearance rate is dictated by the death rate of infected cells and the stage of replication cycle inhibited by the drug and cannot be exceeded with higher doses (126). For SARS-CoV-2, models predicted more rapid initial clearance with higher doses. Yet, after 5 days of treatment, higher doses were predicted to increase the likelihood of virologic rebound by more potently blunting synergistic immune responses (24).

Exploratory simulations can also probe the benefits of narrowing the dosing interval, a potentially useful step when treating viruses with rapid expansion kinetics with short half-life drugs. For instance, simulations of acyclovir for genital HSV-2 infection accurately predicted that dosing multiple times per day would lower viral shedding rate to a greater extent than increasing once daily dosing (26, 281). For HSV-2, the percentage of time that the drug is above the *in vivo* EC50 is a key determinant of efficacy.

Finally, simulations can explore shortening or extending therapies. For hepatitis C, modeling was fundamental to demonstrating that shorter courses of treatment were sufficient to achieve cure (57, 118, 154, 278). For SARS-CoV-2, modeling accurately projected that extending therapy from 5 to 10 days is the most effective method to eliminate viral rebound (24, 278).

## VIRAL ENDPOINT OPTIMIZATION WITH ANTIVIRAL CTS MODELS

Simulations of validated models can explore various virologic endpoints. For SARS-CoV-2 agents, viral clearance slope derived from daily sampling is a more sensitive method than assessing a drop in viral load over 5 days because it factors multiple datapoints (51, 52, 275, 288). Post hoc analysis of modeled data which recapitulates these trial results suggests that post-treatment area under the curve could be an even more sensitive endpoint to detect antiviral activity (42). These observations are useful as they allow equally powered trials with smaller sample sizes. Simulated data are ideal for projecting sample size calculations for future trials.

Selection of optimal endpoints might vary according to drug mechanism of action. Simulations of molnupiravir, which works by inducing lethal mutations into SARS-CoV-2 genomes which are still detected with polymerase chain reaction, suggested that an assay which enumerates only non-mutated genomes would allow for more accurate assessment of drug potency, while current PCR assays underestimate the potency of the drug (42). A similar outcome was observed for HIV integrase inhibitors which induce more rapid viral clearance than other regimens (41, 126). The proposed mechanism is not increased potency, but rather that integrase inhibitors work earlier in the replication cycle compared with protease inhibitors and polymerase inhibitors and thereby prevent the formation of non-infectious genomes which are detectable with PCR. Similar explanations may explain why baloxavir more rapidly eliminates influenza relative to oseltamivir without any apparent additional clinical benefit (197).

## ASSESSING SPECIAL POPULATIONS WITH ANTIVIRAL CTS

Validated models also allow extrapolation of projected results to other study populations. For SARS-CoV-2, untreated viral kinetics shifted with the emergence of new viral variants and of population-level immunity such that duration of viral shedding and peak viral load decreased (51, 288). Therefore, updated simulations were often required to project outcomes of trials under current conditions. For many viruses, including all respiratory viruses, HSV-2, and CMV, viral shedding may be more sustained and at a higher viral load in immunocompromised individuals (267, 285). These conditions favor emergent drug resistance. Yet, trials are difficult to fund in these populations. Modeling is one way to assist treatment guidelines absent clinical trial data. Model formulations may need to include both sensitive and resistant strains and account for dual therapies.

Overall, the validation of a complete antiviral CTS model allows projections of trials under multiple other conditions. This process can de-risk the design of future trials by creating conditions that favor the demonstration of efficacy with the smallest number of participants possible.

## MODELING PRE- AND POST-EXPOSURE PROPHYLAXIS

Because VID models must be validated against longitudinal viral load data, training a model against pre- or post-exposure prophylaxis (PEP) data is difficult, particularly if the intervention completely prevents infection. However, if there has been antecedent validation of a VID model against a separate infection data set, forecasting PEP is possible. Recent models helped explain the failure of SARS-CoV-2 PEP trials as a result of viral rebound due to incomplete elimination of infected cells and virus, as well as short drug half-life (24). Analysis of the Antibody-Mediated Prevention (AMP) trials demonstrated that breakthrough infection with antibody-sensitive HIV was associated with slower expansion slope than breakthrough infection with resistant virus, allowing inference of antibody-mediated pressure against HIV, the deleterious fitness of effects of resistance mutations, and the *in vivo* EC50 for prevention for VRC01, the monoclonal antibody tested in these trials (17, 256). PK and PD modeling of HIV neutralizing antibodies has also been used to estimate the optimal dosing profiles of combinatorial prevention strategies (233). The goal of this work is to translate preventative efficacy to humoral responses elicited by vaccination.

## MODELING SECONDARY PREVENTION

Another area of interest is extrapolating antiviral effects within an individual to the population at large. At a basic level, population models have been used to assess the direct effects of antiviral drugs on reducing hospitalization and death in the larger population (35). Because antivirals reduce viral loads, they may also provide indirect effects by lowering transmission risk. Integration of within-host VID models and epidemiologic transmission models is commonly used to project these effects. The particular viral kinetic profiles and transmission profiles of a given virus are essential to model assumptions. For instance, during generalized HIV epidemics with predominant sexual transmission, most new transmissions occur during the viral load steady state. Therefore, widespread implementation of antiviral therapy profoundly reduces community transmission (289). Yet, early during the COVID-19 pandemic, SARS-CoV-2 viral loads typically peaked before or at symptom onset and then rapidly decreased. Treatment of symptomatic infection was therefore predicted to impact transmission to a much lesser degree, even if implemented widely (290).

## USING ANTIVIRAL CTS TO TRANSLATE PRE-CLINICAL ANIMAL MODELS INTO HUMAN TRIAL OUTCOMES

Preclinical animal models and controlled experimental challenge systems generate longitudinal viral, immunological, and pharmacological data that are critical for mechanistic inference but their value hinges on translation to humans (Fig. 3) (291–294). Mathematical models can distil mechanistic processes from animal data and predict how analogous interventions might perform clinically. Such translation has been demonstrated in influenza (295) and HIV (296). More recently, applied mathematical models were originally used to fit human SARS-CoV-2 clinical trial data to rhesus macaques treated with nirmatrelvir/ritonavir, molnupiravir, or both (67). These models suggested that different drug levels may be required in NHP versus humans to limit viral replication, highlighting limitations of translatability.

## SUMMARY

In this review, we demonstrated that antiviral CTS is useful at multiple stages of the drug development process and is increasingly used to inform specific decisions about drug selections, dosing, dosing interval, timing of treatment, virologic endpoint selection, and trial design. Optimal partnerships between drug development and modeling teams occur throughout the process and include collaborative study design to maximize the utility of data generated for modeling.
